# The NLRP3 inflammasome activation in subcutaneous, epicardial and pericardial adipose tissue in patients with coronary heart disease undergoing coronary by-pass surgery

**DOI:** 10.1016/j.athplu.2022.03.005

**Published:** 2022-03-24

**Authors:** Sissel Åkra, Ingebjørg Seljeflot, Bjørn Braathen, Vibeke Bratseth, Charlotte Holst Hansen, Harald Arnesen, Theis Tønnessen, Svein Solheim

**Affiliations:** a Center for Clinical Heart Research, Department of Cardiology, Oslo University Hospital Ullevål, Oslo, Norway; b Faculty of Medicine, University of Oslo, Oslo, Norway; c Department of Cardiothoracic Surgery, Oslo University Hospital, Oslo, Norway

**Keywords:** Epicardial adipose tissue, Pericardial adipose tissue, Subcutaneous adipose tissue, Coronary heart disease, NLRP3 inflammasome, Interleukin-18, Interleukin-6

## Abstract

**Background and aims:**

Epicardial and pericardial adipose tissue (EAT and PAT) associate with atherosclerosis, however, discussed to have different inflammatory properties. We examined the NLRP3 inflammasome related pathway, playing a pivotal role in atherosclerosis, in EAT, PAT and subcutaneous AT (SAT), their relationship to cell types and anthropometric measures in patients undergoing coronary artery bypass grafting.

**Methods:**

Biopsies from EAT, PAT and SAT were collected from 52 patients with coronary heart disease (CHD) (median body weight 85.0 kg) and 22 controls. RNA was extracted and expression of interleukin (IL)-1β, IL-18, NLRP3, Caspase-1, toll-like receptor 4 (TLR4), IL-6, IL-6 receptor and gp130 were analyzed by RT-PCR.

**Results:**

Limited differences in any genes between CHD patients and controls. IL-18 and IL-6 were 4-fold higher expressed in EAT versus PAT (p < 0.01, both) and SAT (p < 0.001, both), whereas caspase-1, IL-6R and gp130 were higher expressed in SAT compared to the other compartments (all p = 0.06-<0.001). Significant correlations between SAT and PAT gene expressions (r = 0.358–0.579, all p ≤ 0.01). Especially NLRP3 and TLR4 associated with the expression of macrophages in all compartments (all p < 0.001). In EAT IL-18 correlated inversely with the expression of macrophages and T-cells. In SAT and PAT most of the mediators associated with body weight.

**Conclusions:**

Higher expression of IL-18 and IL-6 was observed in EAT in our non-obese CHD patients, not related to inflammatory cells. The NLRP3 inflammasome activation in SAT that mirrored PAT, both related to anthropometrics, suggest that SAT samples, being easily available, to a certain degree, represent adipose tissue inflammation in general.

## Highlights

• Limited differences in NLRP3 inflammasome mediators expressed in SAT, PAT and EAT between CHD patients and controls.
• IL-18 and IL-6 were higher expressed in EAT compared to SAT and PAT in CHD.
• SAT and PAT expression of NLRP3 inflammasome mediators correlated significantly.
• Presence of macrophages and T-cells was more pronounced in SAT and PAT compared to EAT.
• Expression of NLRP3 inflammasome mediators in SAT and PAT, but not EAT, associated with body weight.

## Background

Inflammation plays a central role in the pathogenesis of atherosclerosis, which is the main process causing cardiovascular disease (CVD). It is a key player both in development of the atheroma and in triggering of plaque rupture and thrombus formation [1]. Most of the well-known risk factors for developing atherosclerosis like high blood pressure, insulin resistance and overweight are associated with inflammation. Among these, overweight and obesity is emerging in a global epidemic manner [2].

With excessive energy intake the expansion capacity of the subcutaneous adipose tissue (SAT) is reached and storage of visceral adipose tissue (VAT) occurs to accommodate the overfeeding. Thus, VAT storage can be looked upon as a sign of dysfunctional SAT unable to enlarge [3]. With overweight and obesity the adipose tissue in general change its phenotype from been anti-inflammatory and insulin-sensitive to be pro-inflammatory, pro-thrombotic and insulin resistant, in addition to undergo hypertrophy and increase the release of free fatty acids [4,5]. The adipose tissue is mainly composed of adipocytes, but also monocytes, macrophages, T-cells, fibroblasts and vascular cells are present [6]*.* With overweight and adipose tissue expansion, increased recruitment of pro-inflammatory T-cells and M1 macrophages occur at the expense of anti-inflammatory M2 macrophages [7].

The heart is surrounded by epicardial adipose tissues (EAT) and pericardial adipose tissue (PAT) and these have been claimed to exert different inflammatory properties [8,9], but the amount of both associated with coronary heart disease (CHD) severity [[10], [11]]*.* Studies have also shown both SAT, PAT and EAT to be associated with coronary artery calcium score [12,13]. The EAT, located between the myocardium and the visceral pericardial layer, surrounds the coronary arteries and shares blood flow with the myocardium [4,14]. In normal physiological condition, about 80% of the heart is covered with EAT and it seems to play a protective role, also acting anti-inflammatory [15]. However, with overweight and obesity increased volume of EAT has been associated with atherosclerosis and metabolic syndrome [16], and EAT have therefore been discussed to be a source of circulating inflammatory cytokines [17]. The pericardial adipose tissue (PAT) which is separated from the heart by the pericardium [15], is discussed to have less inflammatory properties, although related to body mass index (BMI), metabolic risk factors and the amount of VAT in some studies [18,19]. A pro-inflammatory state also in SAT has been shown to be associated with CVD [20].

The recent years it has become evident that the NLR family pyrin domain containing-3 inflammasome (NLRP3) is implicated in several disease states and also to play a pivotal role in adipose tissue biology [21]. Activation of NLRP3 is critical for adipose tissue homeostasis and is associated with adipocyte differentiation and adipogenesis both under physiologic conditions and in obesity [22]. We have previously shown that gene expression of the NLRP3 related inflammation in SAT strongly associate with BMI and insulin resistance and with the amount of VAT and SAT, assessed by CT, in a healthy population [23].

The aim of the present investigation was to further explore differences in gene expression and protein secretion of the NLRP3 inflammasome related inflammatory pathway in EAT, PAT and SAT from patients with CHD being exposed during open cardiac surgery. In addition, whether there were any relationship to the cell types expressed in the different compartments, the corresponding circulating markers and to anthropometric measures. Patients in need for valve replacement, without sign of CHD were included as controls.

## Materials and methods

The study was an observational study, conducted in elective patients with CHD undergoing coronary artery bypass surgery with an open chest procedure and the use of extracorporeal circulation. Fifty-two patients with CHD and 22 patients with valve replacement as controls, were included in a period from December 2016 to May 2018 at Oslo University Hospital Ullevål, Oslo, Norway. Written informed consent was obtained from all patients before the surgery. The study protocol was approved by the Regional Ethics Committee of North Norway (# 2016/441), conducted in accordance with the ethical guidelines of the Declaration of Helsinki and is registered at clinicaltrials.gov (NCT02760914). In principle, no restrictions for inclusion and no exclusion criteria were set, however, patients using any medications thought to interfere with inflammation, i.e. steroids a.o., were excluded.

Patients characteristics were obtained the day before surgery, and dietary habits were registered by use of the SmartDiet form, a validated food frequency questionnaire [24]. Each of the items were given a score and summarized to a total score ranging from 15 to 45 points.

During the surgical procedure representative biopsies (approximately 0.5 × 1.5 cm) from EAT, PAT and SAT were collected and carefully processed and snap deep-frozen to −80 °C until RNA extraction for qPCR analyses. EAT was taken from the area between the right coronary artery and the pulmonary artery where appropriate, PAT ventrally to the pericardium in front of aorta, and SAT pre-sternally at the middle of sternum. All samples were collected before starting extracorporeal circulation. Arterial blood samples were collected at start of the anesthesia.

### Laboratory analyses

The selected variables for gene expression were NLRP3, interleukin (IL)-1β, IL-18, Caspase-1, TLR4, IL-6, IL-6 receptor (IL-6R) and gp130. IL-12 was added to underpin whether IL-18 would act in a pro-inflammatory manner. Total RNA was isolated from EAT, PAT and SAT by use of the RNeasy Lipid Tissue Mini Kit according to the manufacturer protocol (Qiagen, GmbH, Hilden, Germany). RNA purity and quantity were measured by the NanoDropTM 1000 Spectrophotometer (Saveen Werner, Sweden). The purity, assessed as the 260 and 280 nm absorbance ratio (260/280) was mean 1.7, and mean quantity was 28,6 ng/μL cDNA was made from equal amount of RNA (5 ng/μL) with qScriptTM cDNA superMix (Quanta Biosciences, Gaithersburg, Maryland, USA). Gene expression analyses were performed with commercially available TaqMan® assays as follows: Interleukin (IL)-6 (Hs00174131_m1), IL-6 receptor (IL-6R) (Hs01075664_m1), gp130 (Hs00174360_m1), NLRP3 (Hs00918082_m1), Caspase 1 (Hs00354836_m1), IL-1β (Hs01555410_m1), IL-18 (Hs00155517_m1), TLR4 (Hs00152939_m1) and IL-12 (Hs01073447_m1), (all Applied Biosystems, Foster City, CA, USA). Details of the assays is available from the commercial source. Real time qPCR was performed on a ViiATM7 instrument (Applied Biosystems) using TaqMan® Universal PCR Master Mix (P/N 4324018). The mRNA levels from the reactions were determined with the ΔΔCT method, normalized to β2-microglobulin (HS99999907_m1) (Applied Biosystems) and related to a reference sample giving relative quantification (RQ) [25]. To determine different cell types present, cluster of differentiation (CD) markers for monocyte/macrophages (CD 163 and CD68), T-cells (CD3), and endothelial cells (CD31) were measured by gene expression of their respective genes.

Serum, obtained by centrifugation within 1 h at 2500×*g* for 10 min were kept frozen at - 80 °C until analysis. Circulating levels of IL-6, IL-6RA, gp130 (all R&D Systems, Inc., 614 McKinley Place NE, Minneapolis, US) and IL-18 (MBL, Medical & Biological Laboratories CO., LTD., Nagoya, Japan) were measured by commercially available enzyme-linked immunosorbent assay (ELISA). The inter-assay coefficients of variation (CV) in our laboratory were 2.7%, 0.8%, 2.6% and 1.4% respectively. Routine analyses were performed by conventional laboratory methods.

### Statistics

Patients characteristics are given as number or proportions and median (25,75 percentiles). The Chi square test was used for differences between groups in categorical variables. As most of the read-out variables were skewed distributed, non-parametric statistics were used throughout, i.e. Mann-Whitney test for group comparisons and Spearmans rho for correlation analyses. For differences between individual compartments, Friedmans test followed by Wilcoxon signed-rank test were used. P-values <0.05 were considered statistically significant, however, Bonferroni correction for multiple comparisons were applied whenever relevant, as described. SPSS version 26 (SPSS Inc., IL, USA) was used throughout.

## Results

Baseline characteristics of the CHD and the control group are shown in Table 1. In the CHD population, 27% had diabetes (23% type 2) and 71.2% were using statins. Blood pressure and routine biochemical variables, including C-reactive protein were within the normal range in both groups.

**Table 1:** Baseline characteristics of the study population. Number (proportions) and median (25, 75 percentiles are given).

	CHD (n = 52)	Control (n = 22)
Age, yrs	66.5 (range 48–82)	69.0 (42–79)
Male/female)	40/12	11/11
Smoker (Current/Previous)	31 (59.6)	10 (45.5)
Previous MI	20 (38.5)	2 (9.1)
Previous PCI	20 (38.5)	0
Hypertension	28 (53.4)	9 (40.9)
Diabetes	14 (26.9)	3 (13.6)
Dyslipidemia	12 (23.1)	3 (13.6)
BMI (kg/m^2^)	27.3 (23.8, 30.3)	28.4 (24,6, 31.6)
Weight (kg)	85.0 (70.2, 95.5)	82.5 (77.5, 107.0)
Waist (cm)	92 (86, 98)	90 (88,101)
SBP (mmHg)	140 (124, 160)	140 (115,164)
DBP (mmHg)	80 (70,87)	79 (70,87)
Total Cholesterol (mmol/L)	3.1 (2.7, 3.4)	2.3 (2.8, 4.6)
HDL-cholesterol (mmol/L)	0.97 (0.75, 1.12)	1.10 (0.87, 1.31)
LDL-cholesterol mmol/mL)	1.8 (1.4, 2.2)	2.2 (1.8, 3.0)
Triglycerides (mmol/mL)	1.2 (1.0, 1.8)	1.0 (0.9, 1.6)
Glucose (mmol/mL)	5.6 (4.9, 6.6)	5.6 (5.0, 6.3)
HbA1c (mmol/mol)	39 (36,51)	36 [33,38]
GFR (%)	90 (75, 95)	80 (68, 912)
Creatinine (mmol/L)	76 (67, 85)	80 (65, 91)
CRP (mg/L)	0.91 (0.49, 1.77)	1.00 (1.00, 2.00)
Medication n (%)		
Aspirin	45 (86.5)	9 (40.9)
Other antiplatelet therapy	14 (26.9)	0
ACEi/ATII	17 (32.7)	9 (40.9)
Betablocker	32 (61.5)	6 (27.3)
Statins	37 (71.2)	0
Insulin	6 (11.5)	0
Antidiabetic drug	11 (21.2)	0
Dietary habits (score)	26.5 [22,31]	27.0 [22,30]

BMI: body mass index; SBP: systolic blood pressure; DBP: diastolic blood pressure; HDL: high density lipoprotein; LDL: low density lipoprotein; HbA1c: glycosylated hemoglobin A1c; GFR: glomerular filtration rate; CRP: C-reactive protein.

All genes were successfully analyzed in all three compartments for all patients, with only a few exceptions in SAT samples, mainly because of failure to analyze (n = 2–4 genes missing). Serum for IL-18, IL-6, IL-6R and gp130 were available in all.

### Difference between CHD and controls

As shown in Supplementary Table 1 the differences in expressed genes between CHD patients and controls were limited. Significantly higher expression of IL-1β and IL-6 in PAT in the CHD-group compared to the control-group were found (p ≤ 0.05, both). NLRP3 showed the same pattern, however, borderline significant (p = 0.062). There were no differences between the groups in corresponding circulating levels, and also not in anthropometric measures or dietary habits. Further analyses have thus, been performed in the CHD group only.

We have previously reported on gene expression of the NLRP3 inflammatory pathway in SAT from healthy, younger subjects [23]. In Supplementary Table 2 we show these to be significantly lower compared to SAT from the present CHD patients.

### Expression of the selected genes in the different compartments

**Fig. 1:**
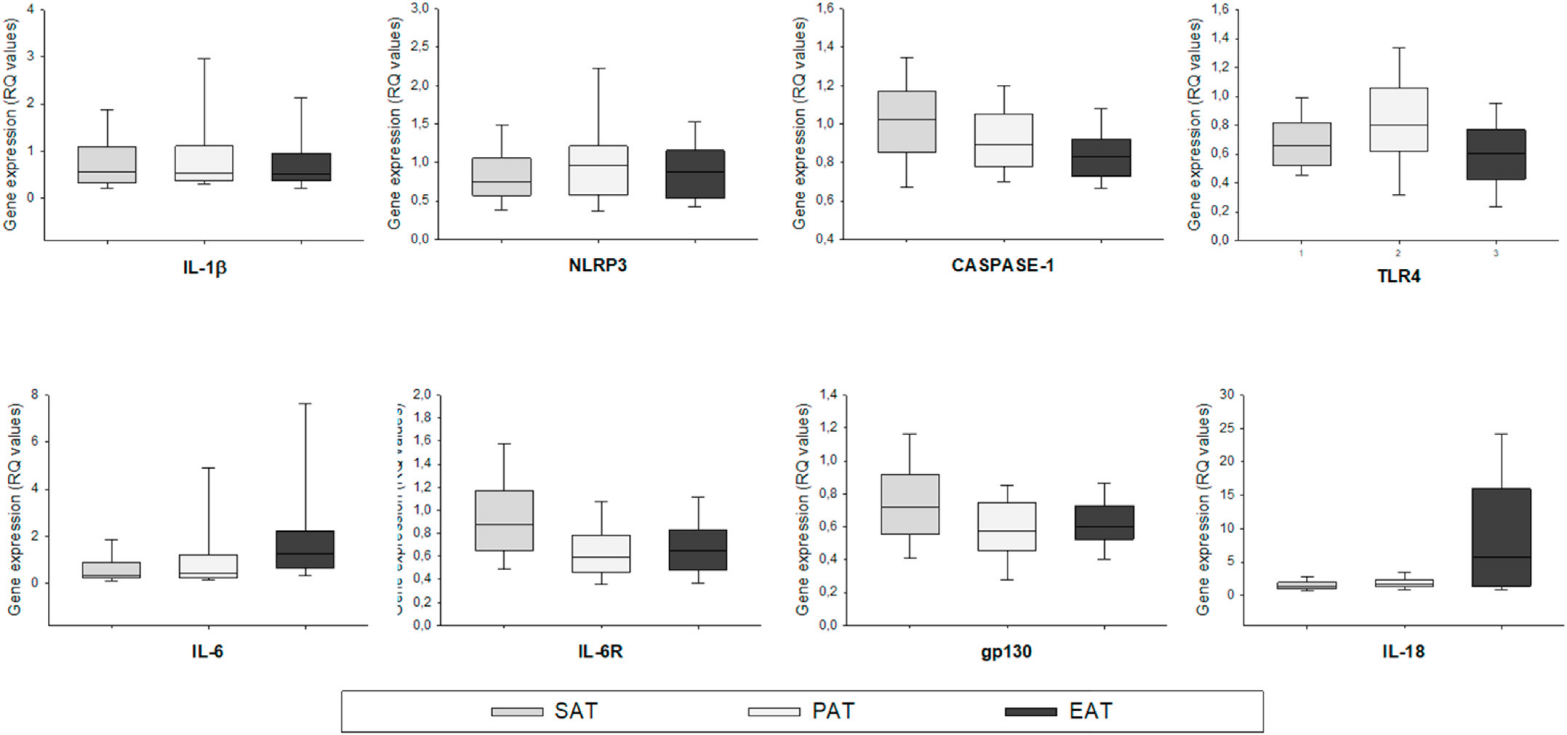
**Expression (RQ values) of the measured genes in the different adipose tissue compartments.** Vertical boxes with median lines and 25,75 percentiles; 10, 90 percentiles indicated by error bars. IL-18 and IL-6 significantly higher expressed in EAT vs SAT (p < 0.001, both) and EAT vs PAT (p < 0.01, both). IL-6R in SAT significantly higher expressed compared to PAT and EAT (p < 0.001, both). gp130 in SAT significantly higher expressed compared to PAT (p = 0.005). Caspase-1 in SAT significantly higher expressed compared to EAT (p < 0.001). TLR4 in PAT significantly higher expressed compared to SAT (p = 0.011) and EAT (p < 0.001). IL-1β and NLRP3 did not differ between compartments.

As shown in Fig. 1 the gene expression of IL-18 and IL-6 showed similar pattern and was the only two genes that were significantly higher in EAT compared to the two other compartments (EAT vs SAT, both p < 0.001) and EAT vs PAT (both p < 0.01). IL-6R was higher expressed in SAT compared to the PAT and EAT (both p < 0.001) and gp130 was higher in SAT compared to PAT (p = 0.005). Caspase-1 showed the highest expression in SAT (vs EAT p < 0.001), whereas TLR4 was highest expressed in PAT (vs SAT p = 0.011, vs EAT p < 0.001). We did not find any differences between any compartments for the expression of IL-1β and NLRP3 (Fig. 1).

### Intra correlations between the measured mediators expressed in the different AT compartments

Genes expressed in SAT and PAT correlated significantly for the following markers: IL-18, IL-1β, NLRP3, IL-6, IL-6R and gp130 (r = 0.375–0.579, all p < 0.01) (Table 2). gp130 expression in SAT correlated also to the expression in EAT (p = 0.001). There was an inverse correlation between IL-18 and IL-12 expression within EAT samples (r = −0.358, p = 0.012), but not within SAT and PAT (Supplementary Table 3a).

**Table 2:** Correlations (Spearmans rho) between the measured mediators expressed in subcutaneous AT versus pericardial and epicardial AT.

	Pericardial	Epicardial
	adipose tissue	adipose tissue
IL-1β	**r** = **0.449**	r = −0.153
	**p** = **0.001**	p = 0.295
IL-18	**r** = **0.375**	r = −0.124
	**p** = **0.008**	p = 0.403
NLRP3	**r** = **0.387**	r = 0.054
	**p** = **0.007**	p = 0.712
Caspase-1	r = 0.358	r = 0.154
	p = 0.120	p = 0.290
TLR4	r = 0.163	r = 0.101
	p = 0.264	p = 0.494
IL-6	**r** = **0.579**	r = 0.041
	**p** ≤ **0.001**	p = 0.777
IL-6R	**r** = **0.409**	r = 0.274
	**p** = **0.004**	p = 0.056
Gp130	**r** = **0.383**	**r** = **0.458**
	**p** = **0.008**	**p** = **0.001**

Abbreviations: See text.

Significant intra correlations between EAT and PAT expressions were present for IL-1β (r = 0.488, p < 0.001), IL-6 (r = 0.595, p < 0.001) and IL-6R (r = 0.416, p = 0.002). In EAT samples there was no significant correlations between IL-6 and IL6R or gp130, whereas within SAT and PAT IL-6 and IL-6R correlated significantly (Supplementary Table 3b).

### Cell markers

**Fig. 2:**
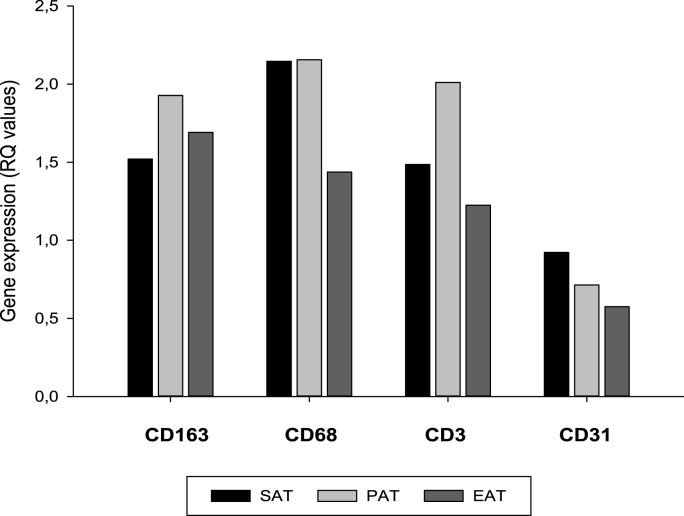
**Distribution of cell markers in the different adipose tissue compartments.** Median RQ values of CD163 and CD68, CD3 and CD31, mainly reflecting monocytes/macrophages, T-cells and endothelial cell, respectively.

Monocyte/macrophages, endothelial cells and T-cells, assessed by their representative CD expression, were present in all tissue samples and the distribution is shown in Fig. 2. Although some differences between the compartments were found, after Bonferroni correction (12 comparisons, i.e. p < 0.004) only the higher CD31 expression in SAT compared to EAT (p < 0.001) was statistically significant.

When relating expression of the inflammatory genes to the different cell types (Table 3), most typical for NLRP3 and TLR4 that correlated especially to CD163 and CD68 in all compartments (all <0.001 after Bonferroni correction (36 comparisons, i.e. p < 0.0014)) (Supplementary Figs. 1a and b). IL-18 expression correlated positively to these cell markers in SAT (Table 3), and of special observation was the inverse correlations in EAT between IL-18 expression and CD163, CD68, CD3 and CD31 (all p ≤ 0.001, corrected) (Table 3, Supplementary Figs. 1c and d). IL-6 and IL-6R expression did not correlate to any cell marker in any compartment, whereas gp130 related to CD68 and CD163, but only in PAT (both p < 0.001 after correction).

**Table 3:**
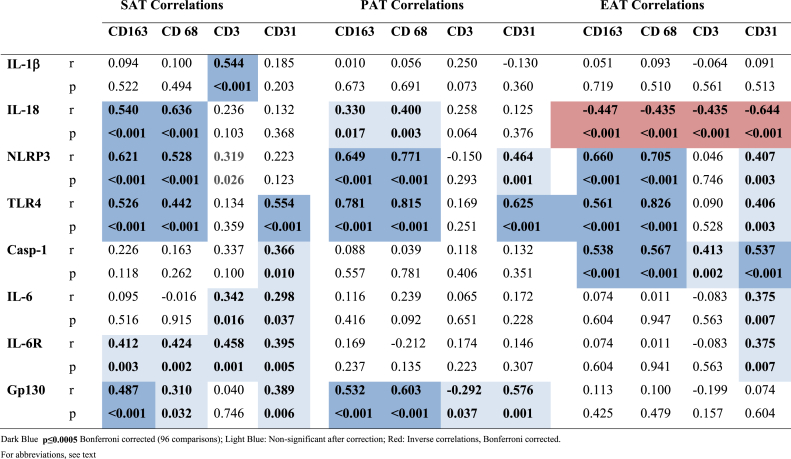
Correlations between theNLRP3 inflammasome variables and markers of cell types in the respective compartments.

### Circulating levels

We found no any significant correlations between circulating levels and their corresponding genes in any AT compartment (Supplementary Table 4), and also no significant associations to anthropometric measures (data not shown).

### Associations between the measured variables and disease entities and anthropometrics

No differences were found in any variable between sex, smoking status, use of statins or the presence of hypertension. In diabetes patients significantly higher levels of circulating gp130 and higher expression of caspase-1 in EAT (p < 0.05, both) were found, and patients with previous myocardial infarction had higher circulating levels of IL-18 (p = 0.020).

**Fig. 3:**
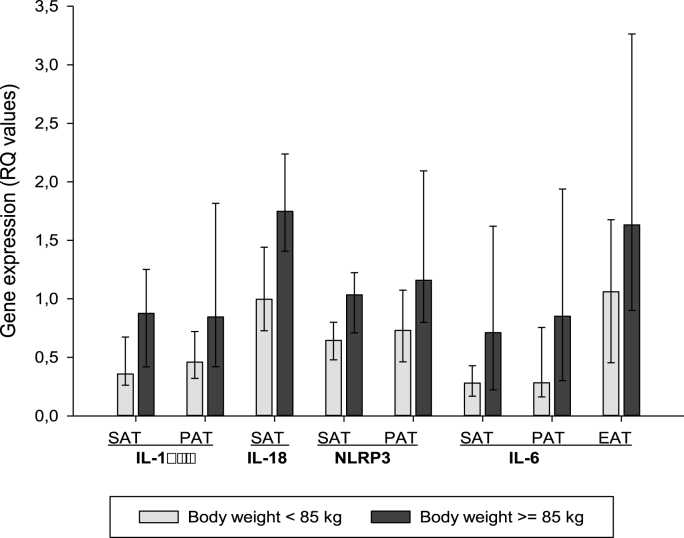
Genes regulated according to body weight below and above median level (85 kg). Bars indicate median values and 25,75 percentiles are indicated by vertical lines. Above median vs below median:. In SAT: higher expression of IL-1β (p = 0.032), IL-18 (p = 0.002), NRLP3 (p = 0.003) and IL-6 (p = 0.027). In PAT: higher expression of IL-1β (p = 0.023), IL-18 (p = 0.050), NLRP3 (p = 0.002) and IL-6 (p = 0.003). In EAT: higher expression of IL-6 only.

In patients with weight above median (85.0 kg) higher expression of IL-1β, IL-18, NLRP3 and IL-6 was found in SAT and PAT compared to the lower weight group (p = 0.002–0.050). IL-6 was higher expressed in EAT only (p = 0.033), in the higher compared to the lower weight group (Fig. 3, Supplementary Table 5). When dichotomizing BMI at median level (27.3 kg/m^2^) a similar picture was seen (data not shown).

There was no significant associations with dietary habits, either analyzing the scores as continuous variables or grouped into below/above median score (data not shown).

## Discussion

Cardiac imaging methodology like coronary computer tomography (CCT) has lately been widely used in order to study the amount and distribution of the different fat compartments as related to CVD [26], and increased EAT and PAT volume has been associated with atherosclerosis and adverse CVD prognosis [12,27]*.* Some studies refer with the term EAT, not distinguishing between EAT and PAT. However, it is now obvious that these have different metabolic and physiologic properties, and should not be used interchangeably (28*).*

Increased inflammation both in EAT and PAT, assessed by high attenuation imaging i.e. CCT and positron emission tomography (PET), have also been shown to associate with coronary atherosclerosis [29,30]. More limited are studies assessing differences between the different locations of adipose tissue with regard to inflammation at a molecular level. To our knowledge, no studies comparing the NLRP3 inflammasome related inflammation in EAT, PAT and SAT in CHD patients have been reported. With the recently shown clinical benefit of inhibition of the NLRP3 pathway on cardiovascular risk, i.e. the CANTOS study with IL-1β antagonism and the ASSAIL-MI study, using IL-6R inhibition [31,32], indicate this pathway to be of great importance. Also other approaches to reduce the inflammatory residual risk in CVD states, including lipid-lowering drugs, that imply the NLRP3 pathways have recently been strongly highlighted [33,34].

The limited differences we found between the CHD patients and the controls may to some degree, be explained by the limited differences in anthropometric and metabolic characteristics between the groups. Although the controls, being aortic valvular disease patients, should be free of CHD, any atherosclerotic burden cannot be ruled out and may thus have masked any difference. Also, use of statins and aspirin, both having anti-inflammatory properties was more frequent in the CHD group. Especially statins have been shown to reduce the volume of EAT [35]. In a recent smaller study, IL-1β and IL-6 were found higher expressed in pericoronary artery adipose tissue, probably comparable with the EAT in our study, in patients with atherosclerotic heart disease compared to controls with mitral valve surgery [36]. This CAD population consisted of a large proportion with diabetes and hypertension, which may explain the difference. We did nevertheless show that the genes were higher expressed in SAT in our CHD population compared to a healthy, younger population [23].

It has been discussed that EAT has the feature to be the most pro-inflammatory compartment, shown by the shifts in their phenotype with atherosclerosis [17,37]. This is in line with our findings of significantly higher expressed IL-18 and IL-6 in EAT, compared to PAT and SAT. Infiltration of inflammatory cells into EAT might to some degree explain the results, despite limited differences in the distribution of cell types between the compartments were found. IL-18 was however, more associated with monocyte/macrophage infiltration in SAT and PAT than in EAT, but a significantly higher proportion of infiltrated endothelial cells was found in EAT, which also, although not typically, can be the source of IL-18 [38]*.* However, surprisingly, IL-18 expression in EAT associated inversely with the presence of monocytes/macrophages, T-cells and endothelial cells. This may be explained by the properties of the macrophages in EAT to be different from other adipose tissue compartments, and infiltrated T cells and macrophages in EAT seems to origin from intravascular inflammatory cells [17]. There may also be more pre-adipocytes in EAT, which have not been differentiated to macrophages [39]. However, as IL-18 correlated inversely also with IL-12 which is needed for the pro-inflammatory action of IL-18 [38], and in EAT only, it may also be speculated whether the results represent an anti-inflammatory state in EAT, thus a part of the protective mechanisms of EAT [28]. In a similar context, the high expression of IL-6 in EAT without any associations with any cell types, and not correlated to IL-6R or gp130, either soluble or at the expression level, may be a sign of the classic IL-6 activation with an anti-inflammatory response [40]. To induce downstream pro-inflammatory responses activation of the *trans*-signaling pathway in which IL-6 bound to the soluble IL-6R activate the membrane-bound gp130, is needed. These suggestions may be speculative, but our population was not obese, thus not with high amount visceral fat, discussed to have similar phenotype as EAT in obese individuals.

None of the other measured markers were higher expressed in EAT compared to PAT and SAT, but interestingly, caspase-1, IL-6R and gp130 were higher expressed in SAT compared to EAT, and TLR4 was higher expressed in PAT compared to the other compartments. Thus, also PAT and SAT presents with pro-inflammatory phenotypes in this population. The strong inter correlations found between SAT and PAT in the expression of almost all the measured genes, with limited or no associations to EAT, indicate similar inflammasome regulation in these compartments, supporting their role in atherosclerosis. The infiltration of pro-inflammatory cells was also highly present in both SAT and PAT, in fact higher than in EAT, again indicating their pro-inflammatory phenotypes. PAT per se has furthermore been shown to be a mediator of metabolic risk and CVD, and thus contribute to coronary atherosclerosis [18,27].

We have previously shown circulating levels of IL-18 to be associated with gene expression of NLRP3 in SAT in a healthy cohort [23], and circulating IL-18 levels were also shown to correlate to IL-18 gene expression in EAT in another study [41]. We could not demonstrate significant correlations between any of the measured circulating markers and their corresponding genes in any adipose tissue compartment. Circulating levels may probably not directly reflect the local adipose tissue inflammation, as also discussed by others [15,17]. In addition, although upregulated, not all genes translate to protein production, and again use of medication could have masked potential associations.

We also observed limited relationship between the measured markers and metabolic factors, somewhat in contrast to our previous findings in healthy individuals [23], demonstrating strong correlations between SAT expression of IL-18 and NLRP3 and glucometabolic variables. Also other studies have showed clear associations between SAT IL-18 expression and insulin resistance and obesity [42,43]. The discrepancy is probably due to differences in the populations with the present CHD population having their comorbidities and use of medications. However, when looking into those with the highest weight, significantly higher inflammatory burden was observed in SAT and PAT, expressed by higher expression levels of IL-1β, IL-18, NLRP3 and IL-6 with higher weight. A similar picture was observed with high and low BMI.

The higher circulating levels of IL-18 observed in patients with a previous myocardial infarction, is consistent with the literature [44,45], although not accompanied by higher expression of the corresponding gene in any adipose tissue compartment.

Although we found limited associations between the measured markers and cardiovascular risk factors in the our population, the NLRP3 inflammasome as biomarker is of great interest, and is a promising tool in improving cardiovascular risk [46,47].

There are several limitations in our study. Being an observational study, only associations and no causality can be explored. As discussed, any degree of atherosclerosis in the control group cannot be ruled out. The CHD patients were optimally treated with a high proportion of aspirin and statin users, and they were not obese as a group. The lack of CT or other imaging measures of adipose tissue amount in the different locations is also a limitation for even more insight into the states of inflammation. We have investigated only the genetic expression of the variables, and as discussed, not all genes are translated to proteins, and the corresponding proteins present have not been investigated. We chose to study the NLRP3 inflammasome pathway, and other inflammatory pathways may act differently. It should also be emphasized that the lack of significance in some of the comparisons could be due to low statistical power rather than to the absence of an actual difference.

The strength of the design with simultaneous collected adipose tissue samples from the two different compartments surrounding the heart, and the subcutaneous adipose tissue, gives the opportunity for specifically studying the separate parts with regard to inflammation at a molecular level.

Taken together, the NLRP3inflammasome was found expressed in all adipose tissue compartments, supporting this pathway to be treatment targets in cardiovascular disease.

IL-18 and IL-6, but not other NRLP3 related mediators, were higher expressed in EAT, compared to PAT and SAT in our non-obese CHD population, and the EAT IL-18 expression correlated inversely with IL-12 and the presence of macrophages, T-cells and endothelial cells, suggesting either other sources or an anti-inflammatory state of the tissue. The results further show that the inflammasome related genes in SAT mirrored that in PAT, both associated with the presence of macrophages, and related to body weight. It may therefore, be suggested that SAT samples, being easily available, to a certain degree, is representative for adipose tissue inflammation in general in non-obese individuals.

## Financial support

We thank Stein Erik Hagens Foundation for Clinical Heart Research, Oslo, Norway for financial support.

## Authorship contribution statement

Sissel Åkra: Conceptualization, acquisition, laboratory and statistical analysis, interpretation of data, original draft and editing. Ingebjørg Seljeflot: Conceptualization, statistical analyses, interpretation of data, original draft and editing. Supervision. Bjørn Braathen: Conceptualization, acquisition, interpretation of data, review and editing. Vibeke Bratseth Conceptualization, acquisition, interpretation of data, review and editing. Charlotte Holst Hansen: Acquisition of data, database management, interpretation of data, review and editing. Harald Arnesen: Conceptualization, interpretation of data, review and editing, Supervision. Theis Tønnessen: Conceptualization, acquisition, review and editing, Supervision. Svein Solheim: Conceptualization, interpretation of data, review and editing. Supervision.

## Declaration of competing interest

The authors declare that they have no known competing financial interests or personal relationships that could have appeared to influence the work reported in this paper.
