# Supplementary material for: The NLRP3 inflammasome activation in subcutaneous, epicardial and pericardial adipose tissue in patients with coronary heart disease undergoing coronary by-pass surgery
Source: Atheroscler Plus. 2022 Mar 24;48:47–54. doi: 10.1016/j.athplu.2022.03.005 (PMC9833236; doi:10.1016/j.athplu.2022.03.005)
Supplement: Multimedia component 1 [file mmc1.pptx]

## Slide 1
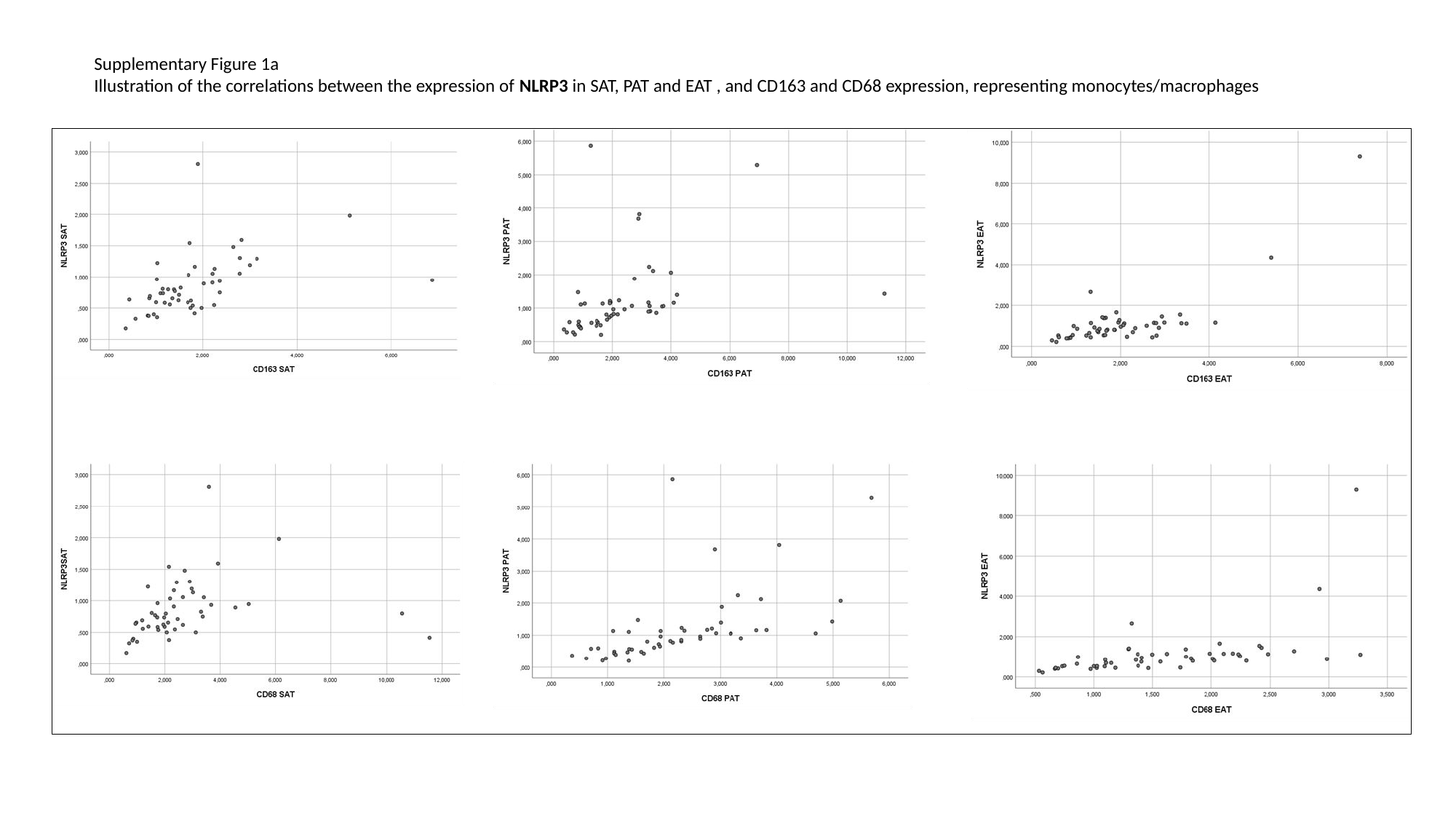

Supplementary Figure 1a
Illustration of the correlations between the expression of NLRP3 in SAT, PAT and EAT , and CD163 and CD68 expression, representing monocytes/macrophages

## Slide 2
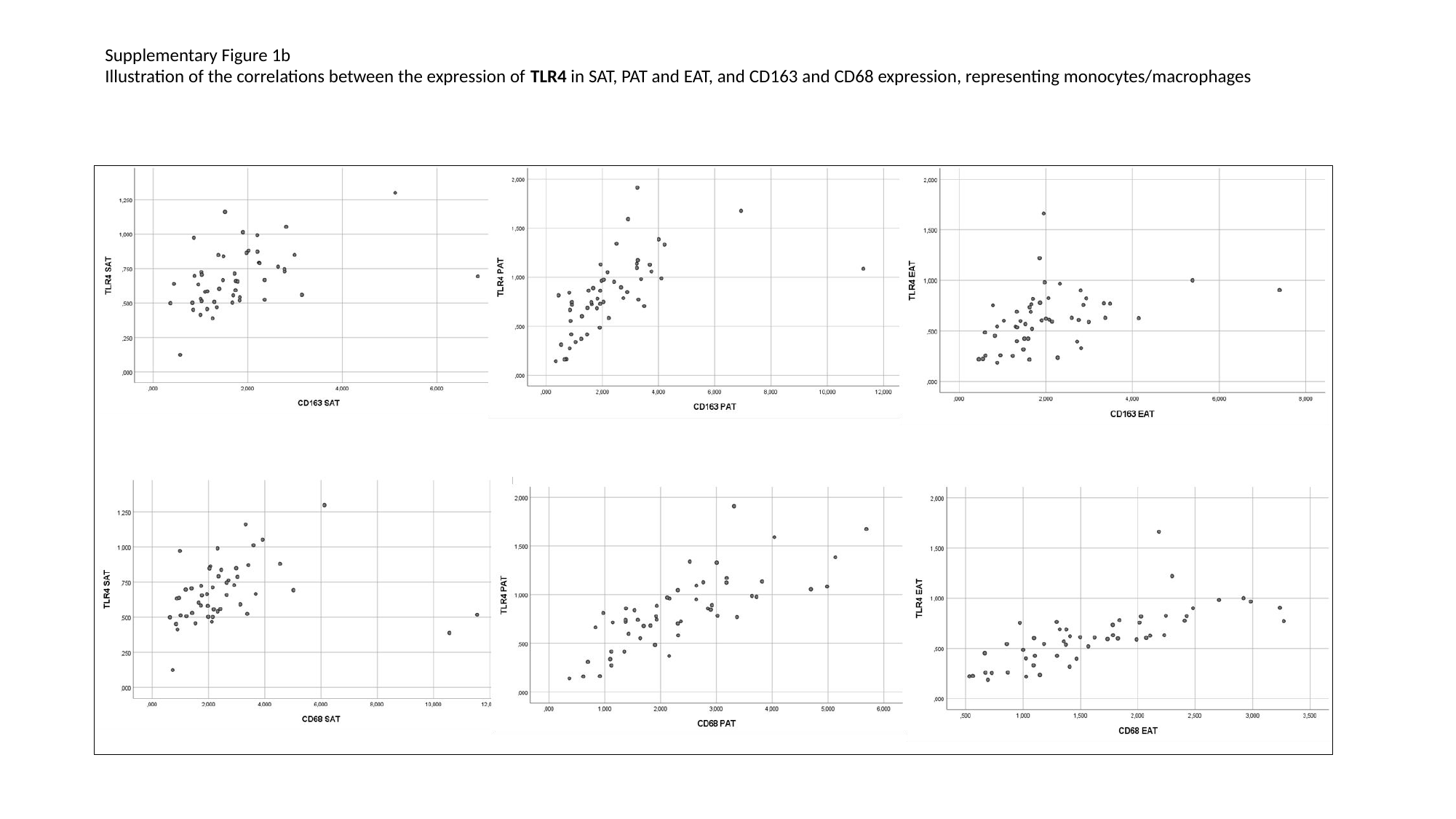

Supplementary Figure 1b
Illustration of the correlations between the expression of TLR4 in SAT, PAT and EAT, and CD163 and CD68 expression, representing monocytes/macrophages

## Slide 3
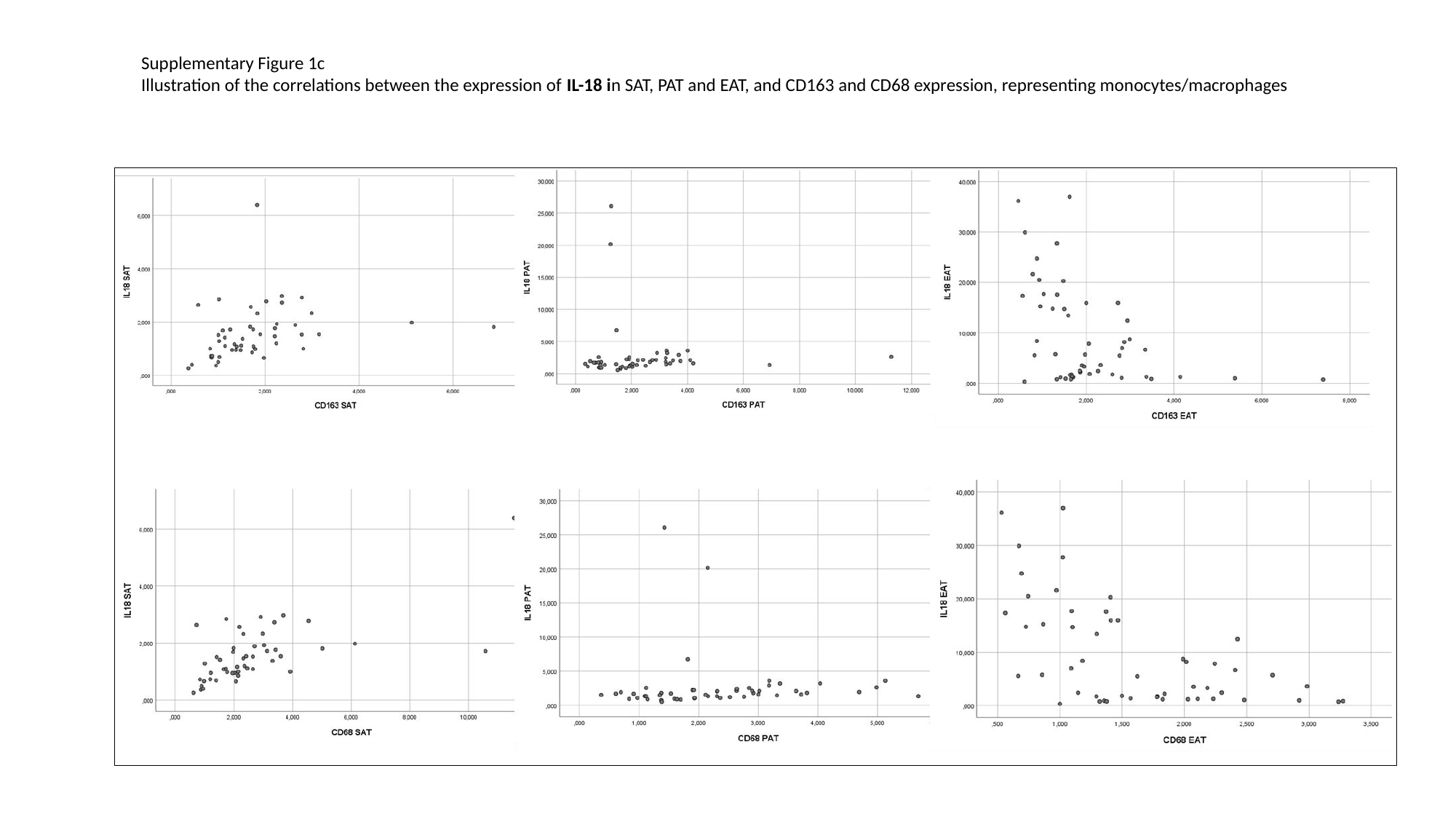

Supplementary Figure 1c
Illustration of the correlations between the expression of IL-18 in SAT, PAT and EAT, and CD163 and CD68 expression, representing monocytes/macrophages

## Slide 4
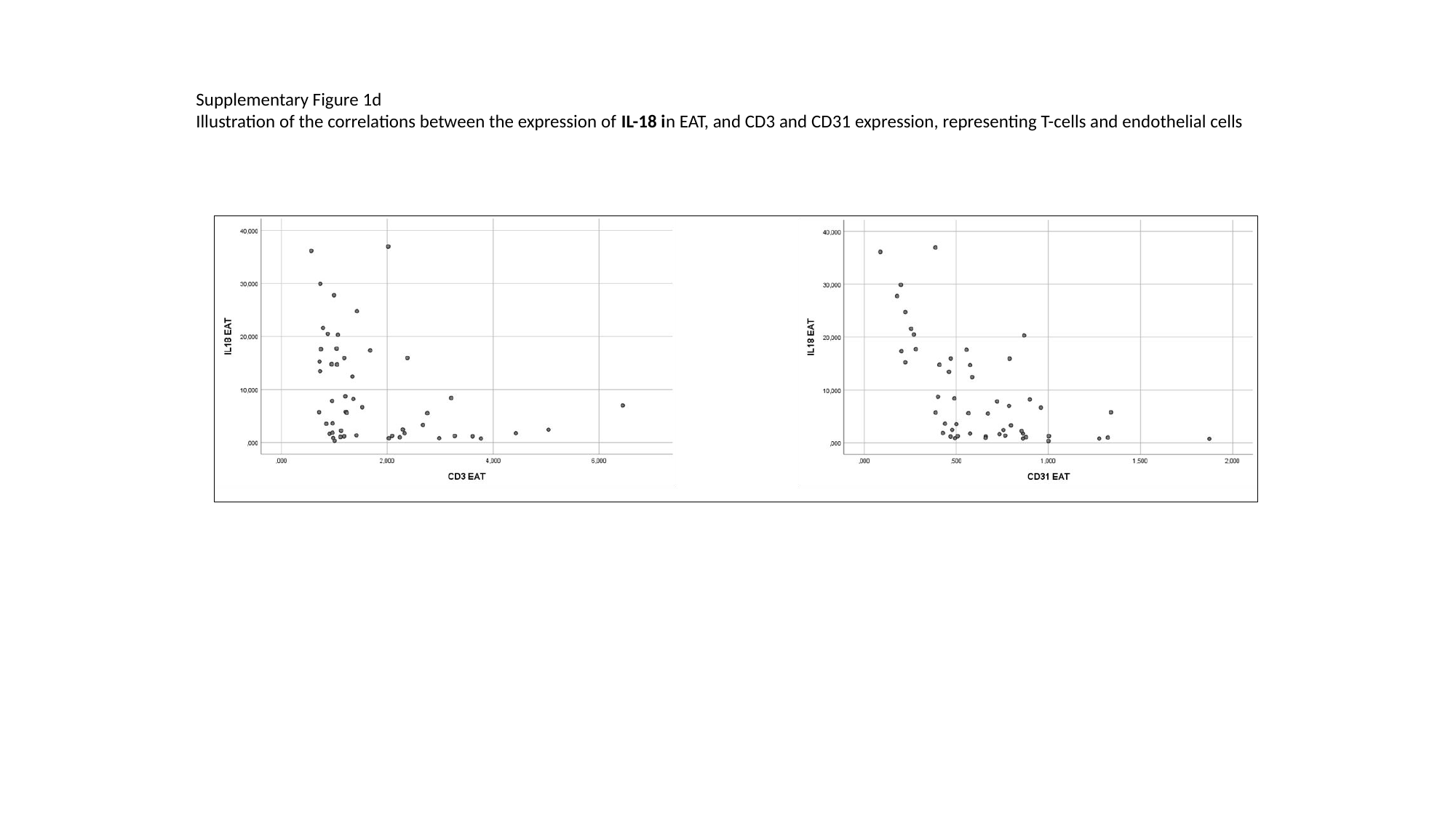

Supplementary Figure 1d
Illustration of the correlations between the expression of IL-18 in EAT, and CD3 and CD31 expression, representing T-cells and endothelial cells
