# Supplementary material for: The NLRP3 inflammasome activation in subcutaneous, epicardial and pericardial adipose tissue in patients with coronary heart disease undergoing coronary by-pass surgery
Source: Atheroscler Plus. 2022 Mar 24;48:47–54. doi: 10.1016/j.athplu.2022.03.005 (PMC9833236; doi:10.1016/j.athplu.2022.03.005)
Supplement: Multimedia component 2 [file mmc2.docx]

**Supplementary Tables 1-5**

**The NLRP3 inflammasome activation in subcutaneous, epicardial and pericardial adipose tissue in patients with coronary heart disease undergoing coronary by-pass surgery**

**Supplementary Table 1.**

Gene expression in the different adipose tissue compartments and corresponding circulating levels in CHD patients (n=52) and controls (n=22)

Adipose tissue Circulating levels

|  | CHD | Control | p | CHD | Control | p |
| --- | --- | --- | --- | --- | --- | --- |
|  |  |  |  |  |  |  |
| IL-1β |  |  |  |  |  |  |
| Subcutaneous | 0.53 (0.31, 1.08) | 0.39 (0.35,0.58) | 0.250 |  |  |  |
| Pericardial | 0.52 (0.33, 1.06) | 0.35 (0.27, 0.44) | **0.021** |  |  |  |
| Epicardial | 0.51 (0.33, 1.06) | 0.44 (0.27, 1.23) | 0.786 |  |  |  |
| IL-18^a^ |  |  |  | 230 (170,332) | 216 (173,271) | 0.347 |
| Subcutaneous | 1.42 (0.95, 1.90) | 1.04 (0.86, 1.43) | 0.102 |  |  |  |
| Pericardial | 1.73 (1.29, 2.24) | 1.88 (1.18, 2.12) | 0.962 |  |  |  |
| Epicardial | 5.70 (1.35, 15.92) | 7.40 (1.64, 17.00) | 0.478 |  |  |  |
| NLRP3 |  |  |  |  |  |  |
| Subcutaneous | 0.75 (0.57, 1.05) | 0.59 (0.48, 0.71) | **0.026** |  |  |  |
| Pericardial | 0.96 (0.57, 1.21) | 0.67 (0.35, 1.03) | 0.062 |  |  |  |
| Epicardial | 0.87 (0.53, 1.15) | 0.66 (0.36, 1.13) | 0.223 |  |  |  |
| Caspase1 |  |  |  |  |  |  |
| Subcutaneous | 1.02 (0.85, 1.17) | 0.95 (0.85,1.17) | 0.905 |  |  |  |
| Pericardial | 0.89 (0.78, 1.05) | 1.00 (0.76, 1.16) | 0.388 |  |  |  |
| Epicardial | 0.83 (0.73, 0.92) | 0.90 (0.80, 0.91) | 0.428 |  |  |  |
| TLR4 |  |  |  |  |  |  |
| Subcutaneous | 0.66 (0.52, 0.82) | 0.64 (0.55, 0.87) | 0.667 |  |  |  |
| Pericardial | 0.80 (0.61, 1.05) | 0.85 (0.44, 1.03) | 0.649 |  |  |  |
| Epicardial | 0.60 (0.42, 0.77) | 0.64 (0.37, 0.82) | 0.714 |  |  |  |
| IL-6^b^ |  |  |  | 2.56 (2.02,3.30) | 2.37 (1.80,2.95) | 0.329 |
| Subcutaneous | 0.34 (0.21, 0.88) | 0.36 (0.20, 0.60) | 0.547 |  |  |  |
| Pericardial | 0.43 (0.22, 1.19) | 0.23 (0.17, 0.40) | **0.035** |  |  |  |
| Epicardial | 1.26 (0.66, 2.23) | 0.72 (0.46, 1.63) | 0.074 |  |  |  |
| IL-6R^b^ |  |  |  | 30740 (24695,36948) | 31645 (23454, 35872) | 0.836 |
| Subcutaneous | 0.87 (0.65, 1.17) | 0.93 (0.69, 1.16) | 0.761 |  |  |  |
| Pericardial | 0.59 (0.46, 0.78) | 0.63 (0.50, 0.80) | 0.656 |  |  |  |
| Epicardial | 0.65 (0.48, 0.83) | 0.60 (0.52, 0.72) | 0,566 |  |  |  |
| Gp130 |  |  |  | 227 (207,242) | 226 (202,254) | 0.953 |
| Subcutaneous | 0.72 (0.55, 0.92) | 0.72 (0.59, 0.97) | 0.783 |  |  |  |
| Pericardial | 0.57 (0.45, 0.75) | 0,53 (0.40, 0.67) | 0.285 |  |  |  |
| Epicardial | 0.60 (0.52, 0.73) | 0.59 (0.50, 0.76) | 0.896 |  |  |  |

p-values refer to difference between CHD and Controls (Mann-Whitney U test)

^a^ng/mL; ^b^pg/mL

Abbreviations: See text

**Supplementary Table 2**

Comparison of gene expression levels (RQ values) in SAT from healthy younger subjects and the CHD patients. Medians and 25,75 percetiles are given.

| SAT  Gene expression | Healthy Young (Ref 21)  (n=109) | CHD  (n=52) | p-value |
| --- | --- | --- | --- |
| IL-18 | 1.46 (0.97,2.18) | 2.57 (1.76,3.48) | <0.001 |
| NLRP3 | 0.58 (0.41,0.94) | 1.52 (1.16,2.17) | <0.001 |
| Caspase-1 | 1.49 (1.29,2.30) | 2.25 (1.87,2.56) | <0.001 |

Abbreviations: See text

p-values refer to difference between CHD and Healthy (Mann-Whitney U test)

**Supplementary Table 3.** Inter correlations (Spearmans rho) between A) gene expression of IL-18 and IL12 and B) between IL-6 and IL-6R and gp130 expression. Significant correlations in bold.

a)

|  | IL-18 SAT | IL-18 PAT | IL-18 EAT |
| --- | --- | --- | --- |
| IL-12 SAT | r=0.062  p= 0.684 |  |  |
| IL-12 PAT |  | r=0.014  p=0.923 |  |
| IL-12 EAT |  |  | **r=-0.358**  **p=0.012** |
|  |  |  |  |

b)

|  | IL-6 SAT | IL-6 PAT | IL-6 EAT |
| --- | --- | --- | --- |
| IL-6R SAT | **r=0.275**  **p=0.050** |  |  |
| IL-6R PAT |  | **r=0.306**  **p=0.029** |  |
| IL-6R EAT |  |  | r=0.091  p=0.527 |
| Gp130 SAT | r=0.164  p=0.481 |  |  |
| Gp 130 PAT |  | r=0.224  p=0.114 |  |
| Gp 130 EAT |  |  | r=0.119  p=0.406 |

**Supplementary Table 4.** Correlations (Spearmans rho) between circulating levels and the corresponding genes expressed in subcutaneous adipose tissue (SAT), pericardial adipose tissue (PAT) and epicardial adipose tissue (EAT)

| Circulating | SAT | PAT | EAT |
| --- | --- | --- | --- |
|  |  |  |  |
| IL-18 | r=-0.07  p=0.961 | r=0.000  p=0.999 | r=-0.027  p=0.849 |
| IL-6 | r=0.011  p=0.940 | r=-0.004  p=0.981 | r=-0.160  p=0.220 |
| IL-6R | r=0-014  p=0.922 | r=0.095  p=0.506 | r=-0.173  p=0.220 |
| Gp130 | r=0.026  p=0.858 | r=-0.105  p=0.465 | r=0.205  p=0.145 |

Abbreviations: See text

**Supplementary Table 5**

Genes expressed in the different compartments according to body weight, dichotomized at median level (85.0 kg)

|  |  | Weight < median | Weight ≥ median | p |
| --- | --- | --- | --- | --- |
| IL-1β | SAT | 0.36 (0.26,0.67) | 0.86 (0.42,1.25) | **0.032** |
|  | PAT | 0.46 (0.32,0.72) | 0.85 (0.42,1.82) | **0.023** |
|  | EAT | 0.49 (0.29, 0.92) | 0.52 (0.40,1.20) | 0.469 |
| IL-18 | SAT | 1.00 (0.73,1.44) | 1.74 (1.41,2.23) | **0.002** |
|  | PAT | 1.63 (1.10,2.24) | 1.96 (1.36,2.41) | **0.050** |
|  | EAT | 3.40 (1.22,15.4) | 7.83 (1.96,16.8) | 0.309 |
| NLRP3 | SAT | 0.64 (0.48,0.80) | 1.03 (0.71,1.22) | **0.003** |
|  | PAT | 0.73 (0.46,1.07) | 1.16 (0.80,2.09) | **0.002** |
|  | EAT | 0.92 (0.46,1.15) | 0.85 (0.53,1.27) | 0.876 |
| Caspase-1 | SAT | 0.94 (0.81,1.19) | 1.05 (0.91,1.14) | 0.542 |
|  | PAT | 0.87 (0.78,1.05) | 0.94 (0.80,1.05) | 0.589 |
|  | EAT | 0.85 (0.72,0.95) | 0.82 (0.73,0.91) | 0.819 |
| TLR4 | SAT | 0.64 (0.50, 0.82) | 0.68 (0.55,0.83) | 0.347 |
|  | PAT | 0.74 (0.67,0.89) | 0.95 (0.53,1.11) | 0.210 |
|  | EAT | 0.60 (0.45,0.77) | 0.59 (0.40,0.77) | 0.692 |
| IL-6 | SAT | 0.28 (0.17,0.43) | 0.71 (0.22,1.62) | **0.027** |
|  | PAT | 0.28 (0.16,0.76) | 0.85 (0.30,2.94) | **0.003** |
|  | EAT | 1.06 (0.45,1.68) | 1.63 (0.90,3.26) | **0.033** |
| IL-6R | SAT | 0.78 (0.58,1.10) | 0.92 (0.71,1.21) | 0.258 |
|  | PAT | 0.58 (0.42,0.73) | 0.66 (0.56,0.89) | 0.069 |
|  | EAT | 0.57 (0.42, 0.73) | 0.71 (0.51, 0.90) | 0.097 |
| Gp130 | SAT | 0.68 (0.52, 0.82) | 0.76 (0.62,0.96) | 0.137 |
|  | PAT | 0.52 (0.41,0.68) | 0.64 (0.48,0.76) | 0.088 |
|  | EAT | 0.56 (0.49,0.66) | 0.63 (0.52,0.80) | 0.089 |

Abbreviations See text

**Supplementary Figure 1**

**Illustrations of the correlations between the expression of NLRP3 (a), TLR4 (b) and IL-18 (c) in SAT, PAT and EAT, and CD163 and CD68 expression, representing monocytes/macrophages.**

Coefficients of correlations (r) and p-values are given in Table 3

**Figure 1d. Illustrations of the correlations between IL-18 in SAT and CD3 and CD31expression, representing T lymphocytes and endothelial cells.**

Coefficients of correlations (r) and p-values are given in Table 3
